# A Size-Controlled Graphene Oxide Materials Obtained by One-Step Electrochemical Exfoliation of Carbon Fiber Cloth for Applications to In Situ Gold Nanoparticle Formation and Electrochemical Sensors—A Preliminary Study

**DOI:** 10.3390/bios12060360

**Published:** 2022-05-24

**Authors:** Jen-Lin Chang, Chen-Wei Liao, D. Arthisree, Annamalai Senthil Kumar, Jyh-Myng Zen

**Affiliations:** 1 Department of Chemistry, National Chung Hsing University, Taichung 402, Taiwan; mistral6886@msn.com (J.-L.C.); way691001@gmail.com (C.-W.L.); 2 Nano and Bioelectrochemistry Research Laboratory, Carbon Dioxide Research and Green Technology Centre, Vellore Institute of Technology, Vellore 632 014, India; arthisree.lakshmi20@gmail.com; 3 Department of Chemistry, School of Advanced Sciences, Vellore Institute of Technology, Vellore 632 014, India

**Keywords:** electrochemical exfoliation, carbon fiber, size-controlled synthesis, graphene oxide quantum dots, fluorescent carbon nanoparticles, electroanalytical applications

## Abstract

A simple, one-step and facile method has been introduced to prepare fluorescent and electrochemically active carbon nanoparticles with single-size distribution and good long-term stability by electrochemical exfoliation of polyacrylonitrile-based carbon fibers in an alkaline solution-phase condition. The preparation condition was systematically optimized by studying the effect of temperature and electrolytes. It has been found that an electrochemical exfoliation reaction carried out at an applied potential of 2 V vs. Ag/AgCl in a phosphate-ion-containing alkaline solution at a temperature of 40 °C is an ideal condition for the preparation of 14 ± 4 nm-sized carbon nanoparticles. Unlike the literature protocols, there are no filtration and membrane dialysis-based off-line sample pretreatments adopted in this work. The as-prepared carbon nanoparticles were characterized by fluorescence, Raman spectrum, transmission electron microscope, and X-ray photoelectron spectroscopic characterization methods. It was found that the carbon–oxygen functional group rich in graphene–oxide quantum dots (GOQDs) such as carbon nanoparticles were formed in this work. A preliminary study relating to simultaneous electrochemical oxidation and the sensing of uric acid and ascorbic acid with well-resolved peaks was demonstrated as a model system to extend the new carbon material for electroanalytical applications. Furthermore, in situ synthesis of 2 nm-sized gold nanoparticles stabilized by GOQDs was presented. The carbon nanoparticles prepared by the direct method in this work have shown good stability over 6 months when stored at room temperature. The electrochemical exfoliation reaction has been found to be highly reproducible and suitable for bulk synthesis of luminescence-effective carbon nanoparticles to facilitate fundamental studies and practical applications.

## 1. Introduction

The allotropes of carbon, such as fullerenes, carbon nanotubes, mesoporous carbon, graphene, and recently graphene quantum dots (GQD), have received tremendous interest in the interdisciplinary areas of material science, engineering, and technology applications. These materials have found applications in electronics, sensors, catalysis, drug delivery, composites, batteries, and so forth [1,2,3,4,5]. As some of the applications require functionalization of their inert graphitic surface, treatment of these materials in oxidizing agents and concentrated acids becomes inevitable. An immediate advantage that comes from such surface functionalization is the dispersibility of the material in water, which is a prerequisite to carrying out all biological applications [6]. From a more intriguing standpoint, it is well known that individual fluorescent and electrochemiluminescence particles have been subjected to the size of graphene-like materials (GQDs) of nearly 2 nm [7,8]. In general, the chemical reaction of graphite by Hummer’s method and hydrothermal-based chemical synthesis routes using precursors such as carbohydrate and biomass samples are recommended for the carbon nanostructures’ graphitic systems [9]. Other approaches involve the exfoliation of carbon from a graphitic source (Hummer’s method), and its dissolution as hydrophilic carbon nanoparticles (CNP) has been predominantly explored [10,11,12,13,14,15,16,17,18,19,20,21,22,23,24,25]. Note that multiwalled carbon nanotubes (MWCNT) were used as a precursor to convert into graphitic materials through an electrochemical method by cycling between −2.0 and 2.0 V versus Ag/AgClO_4_ at a scan rate of 0.5 V/s in a degassed acetonitrile solution with 0.1 M of tetrabutylammonium perchlorate as the supporting electrolyte [22,23]. In all those cases, it is necessary to perform post-chemical treatments of the bulk carbon nanoparticles’ precursors using filtration, centrifugation, and membrane dialysis procedures in order to achieve uniform-sized graphene materials. Thus, direct synthesis of size-controlled graphitic materials without aggregation is a challenging task.

**Scheme 1:**
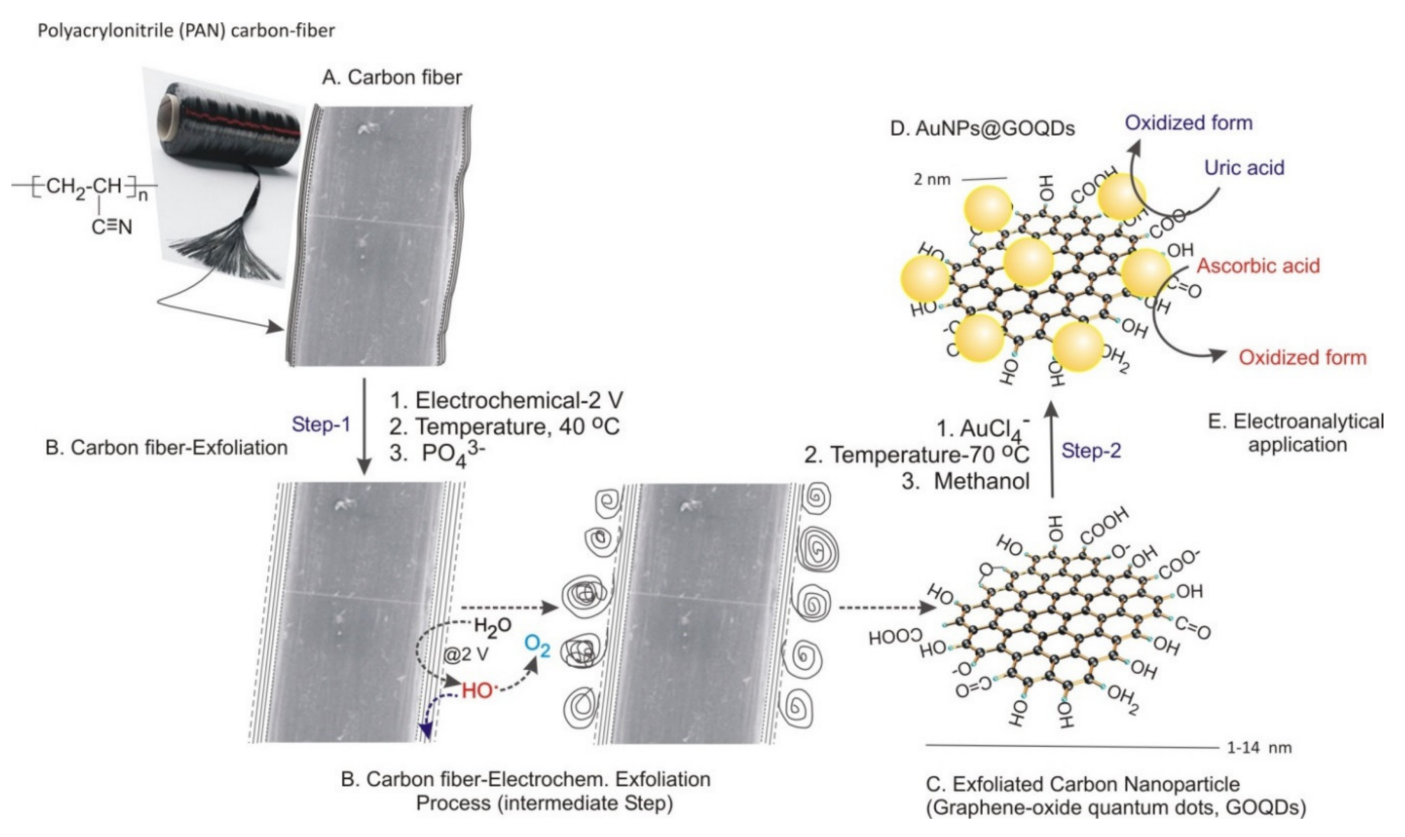
Schematic representation of electrochemical exfoliation of carbon fiber into GOQDs ((**A**,**B**), Step 1) and in situ gold-nanoparticle stabilized GOQDs ((**C**,**D**), Step 2). (**E**) Electroanalytical application for simultaneous oxidation of uric acid (UA) and ascorbic acid (AA).

In this work, we report a simple one-step electrochemical procedure to synthesize homogeneous graphene oxide quantum dots (GOQDs) that have been exfoliated from a carbon cloth made of polyacrylonitrile (PAN) fiber in a sulphate-ion-containing alkaline solution without any specific off-line purification and separation procedures (Scheme 1A–C). The “as-prepared GOQDs water dispersion system” has shown superior long-term stability without the need for any polymeric or surfactant stabilizers or gelators in the medium [23,26,27]. Meanwhile, our group recently reported a pre-anodization condition (potential of 2.0 V versus Ag/AgCl) based on the generation of surface-bound carbon–oxygen functional groups with the generation of edge plane sites on a screen-printed carbon electrode (SPCE) surface and its superior electrocatalytic activity [28,29]. We expect that by applying the same pre-anodization potential under alkaline conditions, exfoliation of graphitic nanoparticles such as GOQDs will be generated from the carbon fiber source. Exfoliated GOQDs that carry a negative charge under alkaline conditions can prevent the particle from aggregating. The direct dispersion of hydrophobic graphitic structures in water, generally considered unattainable, is of great significance in the material science area. A successful formation of relatively pure dispersion enables the use of conventional low-cost, solution-phase processing techniques to create homogenous carbon–carbon nanoparticles (size, 1–14 µm), and for advanced electrochemical applications [1,9]. As a proof of concept, a preliminary electroanalytical study of the simultaneous oxidation and sensing of uric acid (UA) and ascorbic acid (AA), and in situ gold nanoparticle formation aided by GOQDs was demonstrated.

## 2. Experimental Sections

### 2.1. Chemicals and Apparatus

All compounds were American Chemical Society (ACS)-certified reagent grade and used without further purification. The aqueous solution was prepared with Millipore deionized water throughout this investigation. A 0.1 M phosphate buffer solution (PBS) was adjusted with various pH values by using 0.01 M of NaOH and 0.1 M of H_3_PO_4_ solutions for use in the electrochemical synthesis of all nanostructured carbon particles.

### 2.2. Instrumentation

Cyclic voltammetric (CV) and chronoamperometric experiments were performed using a CHI 703 electrochemical workstation (CH Instruments, USA). The polyacrylonitrile carbon fiber cloth was acquired from Taiwan Carbon Technology Co., Ltd. (Taichung, Taiwan). The SPCE with a working area of 0.2 cm^2^ and a conductive track radius of 2.5 mm was purchased from Zensor R&D (Taichung, Taiwan). The surface morphology of the film was examined with a high-resolution transmission electron microscope (HRTEM, EM902 A, Zeiss) operating at 80 kV, and a Topcon ABT-105 instrument (Japan) served as the scanning electron microscope (SEM). A HITACHI U-3000 instrument was used for the UV–Vis measurement. Fluorescence spectra were performed using a HORIBA JOBIN-YVON FluoroMax-4 spectrofluorometer. X-ray photoelectron spectroscopy (XPS) measurements were carried out using an Omicron DAR400, a German instrument with an Al Kα X-ray radiation source (1486.6 eV) with 0.1 eV of resolution. For the XPS deconvolution analysis, th XPSPEAK41 software was used. Room temperature Raman spectra were recorded with a 3D nanometer-scale Raman PL microscopy system by using a Nanofinder^®^ 30 (Tokyo Instruments), a He/Ne laser beam with an excitation wavelength of 488 nm, and a CCD detector (Andor DU401-BV) with a readout speed of 1–32 ls/pixel at −70 °C to record the Raman scattered light intensity. FTIR experiments were carried out using the JASCO FT-IR4100, a Japan instrument. The size distribution of nanostructured carbon particles of each sample solution was measured by a dynamic light scatter (Zetasizer Nano ZS, Malvern Instruments Ltd., UK). Screen-printed carbon electrodes (SPCEs), purchased from Zensor R&D, Taiwan, were used for the electroanalytical studies. The electrode provides a 3 mm diameter-conducting carbon-ink-modified polypropylene-based system.

### 2.3. Preparation of Carbon Nanoparticles

Electrochemical exfoliation experiments were carried out using a piece of carbon fiber cloth (10 mm × 40 mm) immersed in an electrochemical cell containing 0.01 M of NaOH electrolytes with and without phosphate ions at an applied potential of 2.0 V versus Ag/AgCl at different temperatures for 1.5 h. The electrochemical cell consisted of a carbon cloth as a working electrode, a Pt wire as the auxiliary electrode, and an Ag/AgCl reference electrode. The temperature control during the exfoliation process was attained using a through-flow cooler.

### 2.4. Synthesis of Gold Nanoparticles Stabilized by GOQDs

The as-prepared GOQDs were used as a template to fabricate metallic nanoparticles. A gold nanoparticle of average diameter size, ~2 nm of stabilized GOQDs, was synthesized by in situ chemical reduction of HAuCl_4_ using methanol in the presence of the CNP (Scheme 1E). In a typical procedure, GOQDs and the HAuCl_4_ solution were mixed in a volume ratio of 1:5, followed by the slow addition of the same volume of 24.74 M of methanol in a reaction vessel. Then, the reaction mixture was heated at 70 °C for 30 min with constant stirring. The AuNPs@GOQD-modified electrode was prepared by drop-casting 9 µL of the above solution on a cleaned electrode, followed by air drying for ~5 min.

## 3. Results and Discussion

### 3.1. Electrochemical Exfoliation of Carbon Fiber

**Figure 1:**
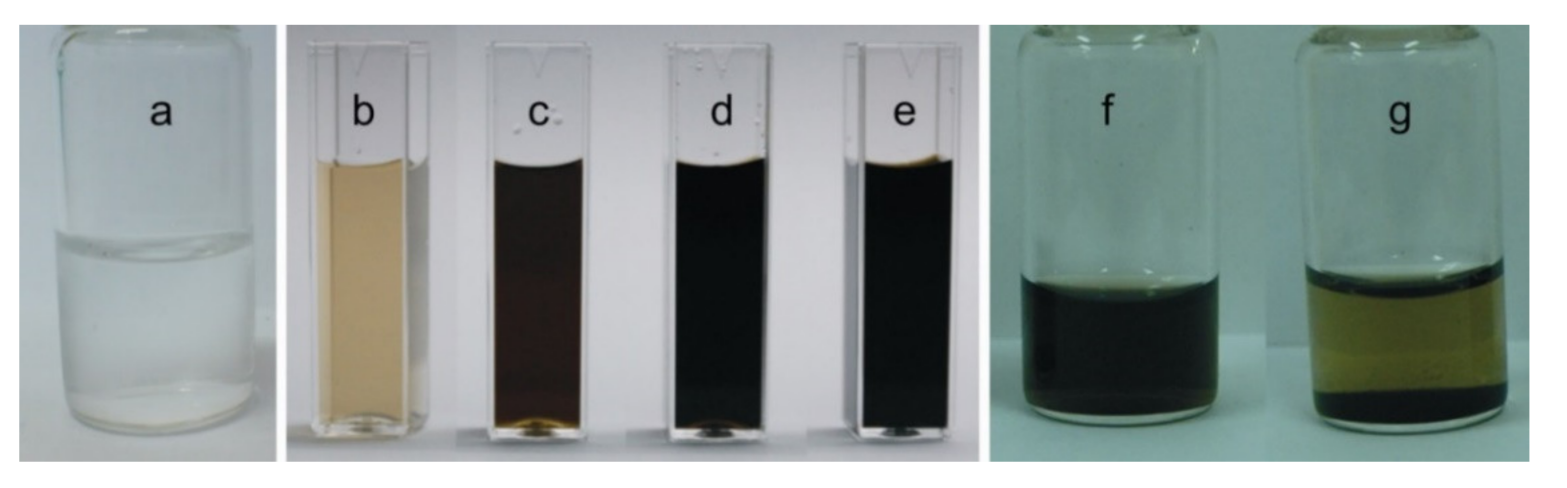
Photographs of electrolyte solutions before (**a**) and after the electro-exfoliated process in 0.01 M of NaOH solution and conditioned at various temperatures of (**b**) 0 °C, (**c**) 20 °C, (**d**) 30 °C, and (**e**) 40 °C for 3. Photograh (**f**) is the “e” stored at 6 months, and after adjusting into acidic condition (pH = 3) (**g**).

Initial electrochemical experiments were carried out using 0.01 M of NaOH electrolyte at different temperatures of 0, 20, 30, and 40 °C. The as-prepared solutions were subjected to various physicochemical and spectroscopic characterizations. Figure 1a–e are the photographs of the electrolyte before (a) and after the electrochemical exfoliation reaction of carbon fiber prepared at different temperatures of 0, 20, 30, and 40 °C in 0.01 M of NaOH solution (b–e). At a low temperature (0 °C), a light blackish solution was noticed, while a dark-colored solution was noticed at high temperatures such as 30 and 40 °C (Figure 1b–f). This observation indicates a relatively higher electrochemical carbonization reaction in a high-temperature system than in the low-temperature preparation condition. Interestingly, when the sample prepared at 40 °C was kept at room temperature for 6 months, an unaltered and stable dispersion was noticed (Figure 1g). This observation is true for other samples, highlighting the formation of highly hydrophilic CNPs in this work. At this stage, it is difficult to predict what kind of CNPs like graphene (Gr), graphene oxide (GO), graphene quantum dots (GQD), and graphene oxide quantum dots (GOQD), etc. formed in this work. To conclude the true carbon nanoparticle, as-prepared samples were subjected to various characterizations.

### 3.2. Physicochemical Characterizations

**Figure 2:**
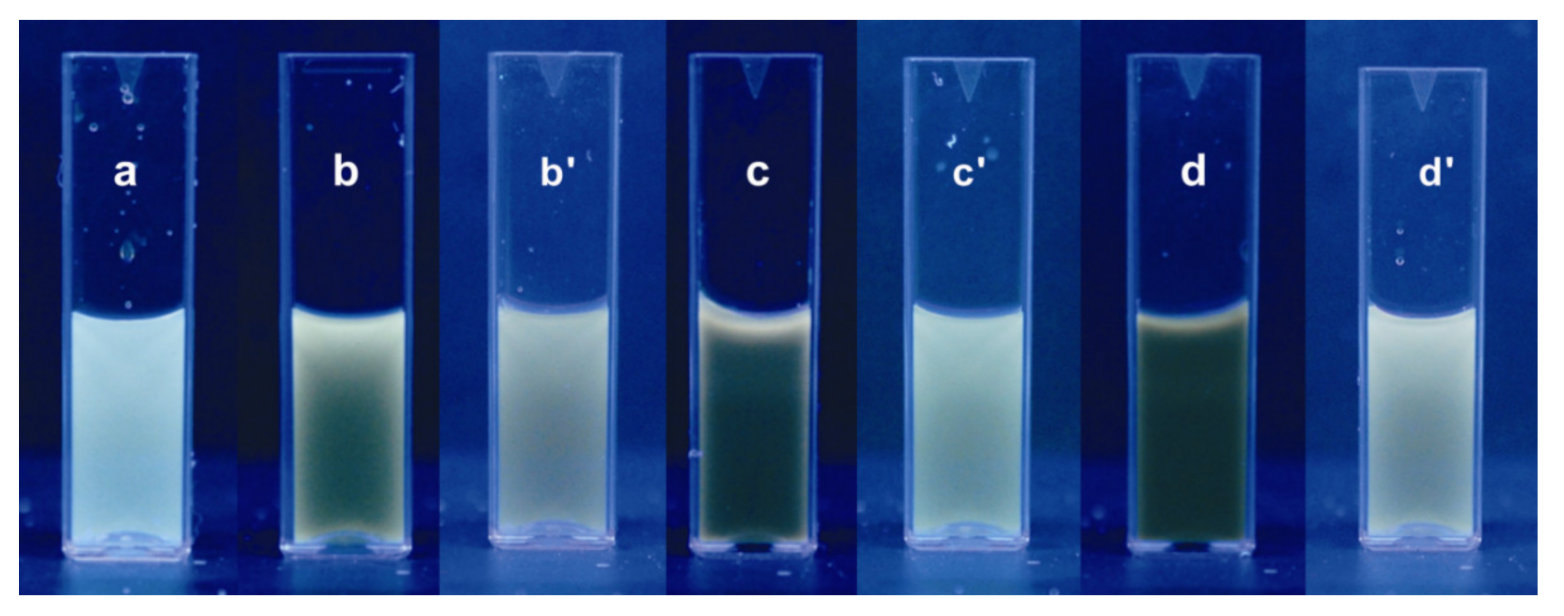
Photographs of the fluorescence of CNP solutions obtained by the electrochemical exfoliation reaction at temperatures of (**a**) 0 °C, (**b**) 20 °C, (**c**) 30 °C, and (**d**) 40 °C. Photographs of (**b’**), (**c’**) and (**d’**) are 4, 8, and 16 dilutions of the respective solutions.

**Figure 3:**
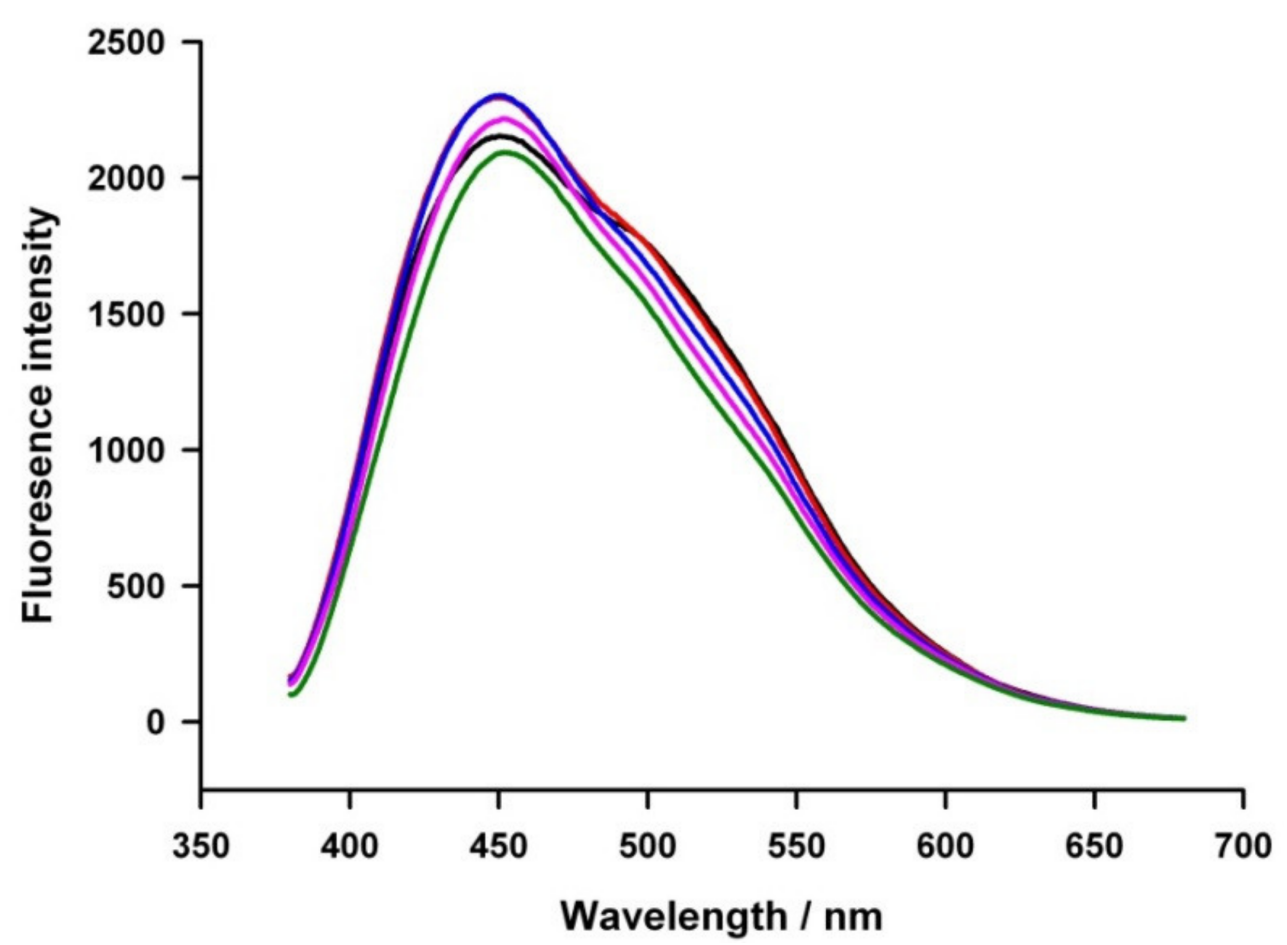
Emission spectra of CNP solutions (40 °C Preparation) at different excitation wavelengths of 310 nm (black line), 320 nm (red line), 330 nm (blue line), 340 nm (pink line), and 350 nm (green line), respectively.

Figure 2a–d are fluorescence responses of carbon nanoparticles prepared at different temperatures and some of their water-diluted samples. Electronic energy transitions like σ*→n (~460 nm)*,* π*→π (520 nm)*,* and π*→n (630 nm) of the sp^3^ and sp^2^ carbon and its oxygen-bonded structures are responsible for the fluorescence observation of carbon nanomaterial [30]. It is likely that both the σ*→n and π*→π transitions were merged and showed an overlapped signal (Figure 3). Furthermore, the carbon nanoparticle system prepared at 40 °C was subjected to fluorescence measurement at different excitation wavelengths of 310, 320, 330, 340, and 350 nm (Figure 3). The emission spectrum of CNPs was found to be excitation-independent. Plausibly, the homogeneous-distribution nature of the solution is the likely reason for the observation. Without any filtrations, good fluorescence spectra of the CNP solutions that achieved blue fluorescence indicated a narrow-sized carbon nanoparticle formation in this work.

**Figure 4:**
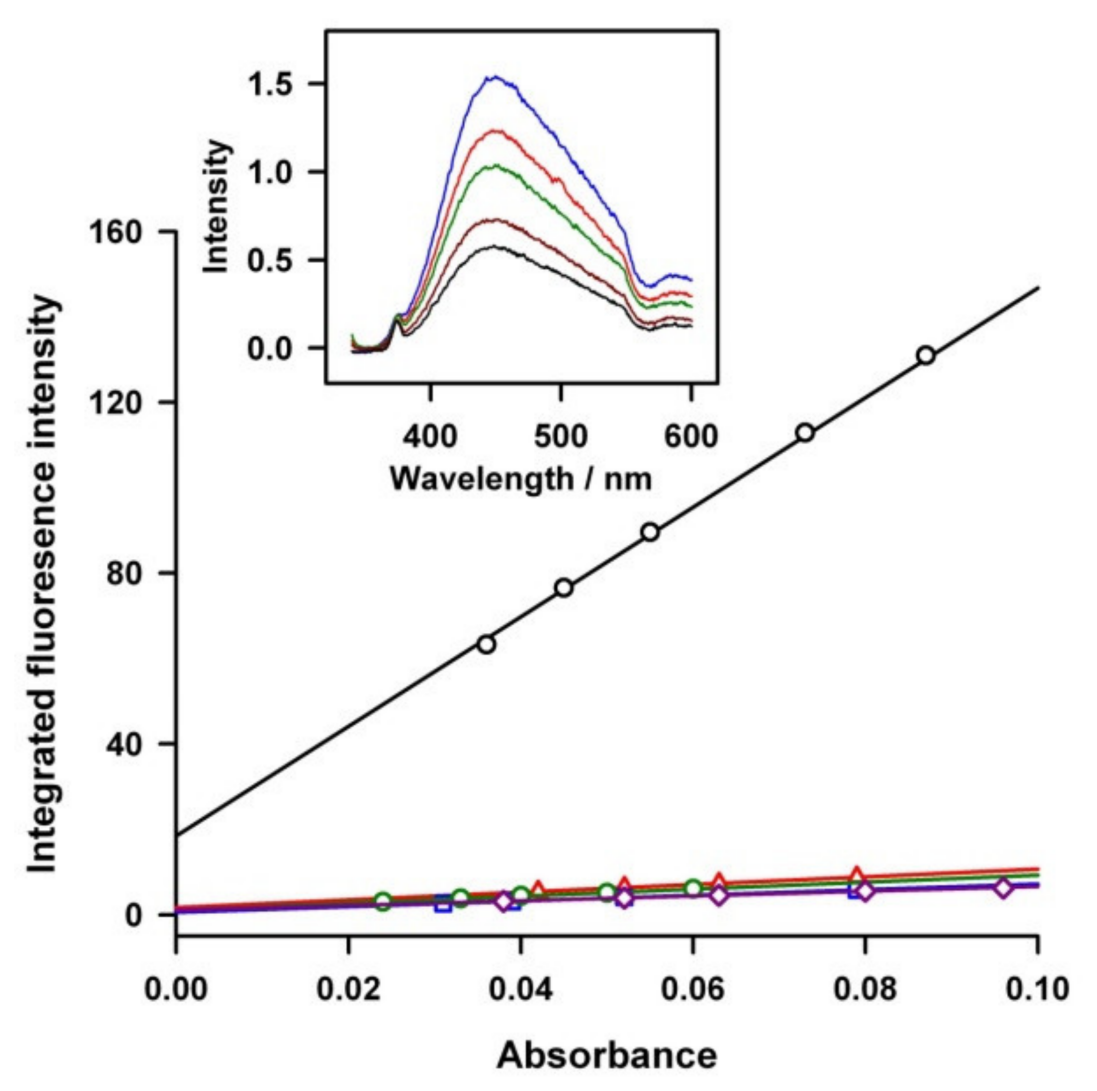
Plots of integrated photoluminescence intensity vs. absorbance of the various nanosized carbon particles, and quinine sulphate (black circle) (diluted samples). The red triangle, blue square, green circle, and purple diamond symbols are the CNPs of electro-exfoliated samples prepared at temperatures of 0 °C, 20 °C, 30 °C, and 40 °C, respectively. The inserted graph is a fluorescence emission spectra of CNPs (average diameter of 1 nm) with various dilution ratios.

A quantum yield (Φ) on the luminescence of the carbon nanoparticle was calculated by comparing the integrated photoluminescence (PL) intensities (excited at 330 nm) and the absorbency values (at 330 nm) of the carbon nanoparticles with the references of quinine sulfate and anthracene, as shown in Figure 4. Five concentrations of each compound were made, all of which had an absorbance of less than 0.1 at 330 nm. Quinine sulfate (literature Φ = 0.54) was dissolved in 0.1 M of H_2_SO_4_ (refractive index (η) of 1.33) and used as a standard solvent, and the carbon sample was dissolved in water (η = 1.33). A UV–Vis absorption spectrometer (HITACHI U-3000) was used to determine the absorbance of the samples at λ = 330 nm. In this study, the respective solvents were used as references. A quartz cuvette with a path length of 1.00 cm was used to carry out the UV–Vis and PL experiments. The PL peaks data were integrated using a WaveMetric Igor program. The absolute fluorescent quantum yield was determined through the following equation: Φ*_x_* = Φ_st_(*m_x_*/*m*_st_) (*η_x_*^2^/*η*_st_^2^) [31] where the subscripts st and *x* denote standard and test samples, respectively, Φ is the fluorescence quantum yield, *m* is the gradient from the plot of integrated fluorescence versus absorbance, and *η* is the refractive index of the solvent. The Φ values were determined to be 0.038, 0.028, 0.034, and 0.023 for exfoliation temperatures of 0 °C, 20 °C, 30 °C, and 40 °C, respectively. These values indicate a homogeneous size distribution of carbon nanoparticles with their controlled diameter. Note that the emission peak of the CNPs did not shift with varying excitation wavelengths, envisaging the homogenous solution nature of the CNPs. The CNP solutions also showed good photostability without using any off-line filtration steps.

**Figure 5:**
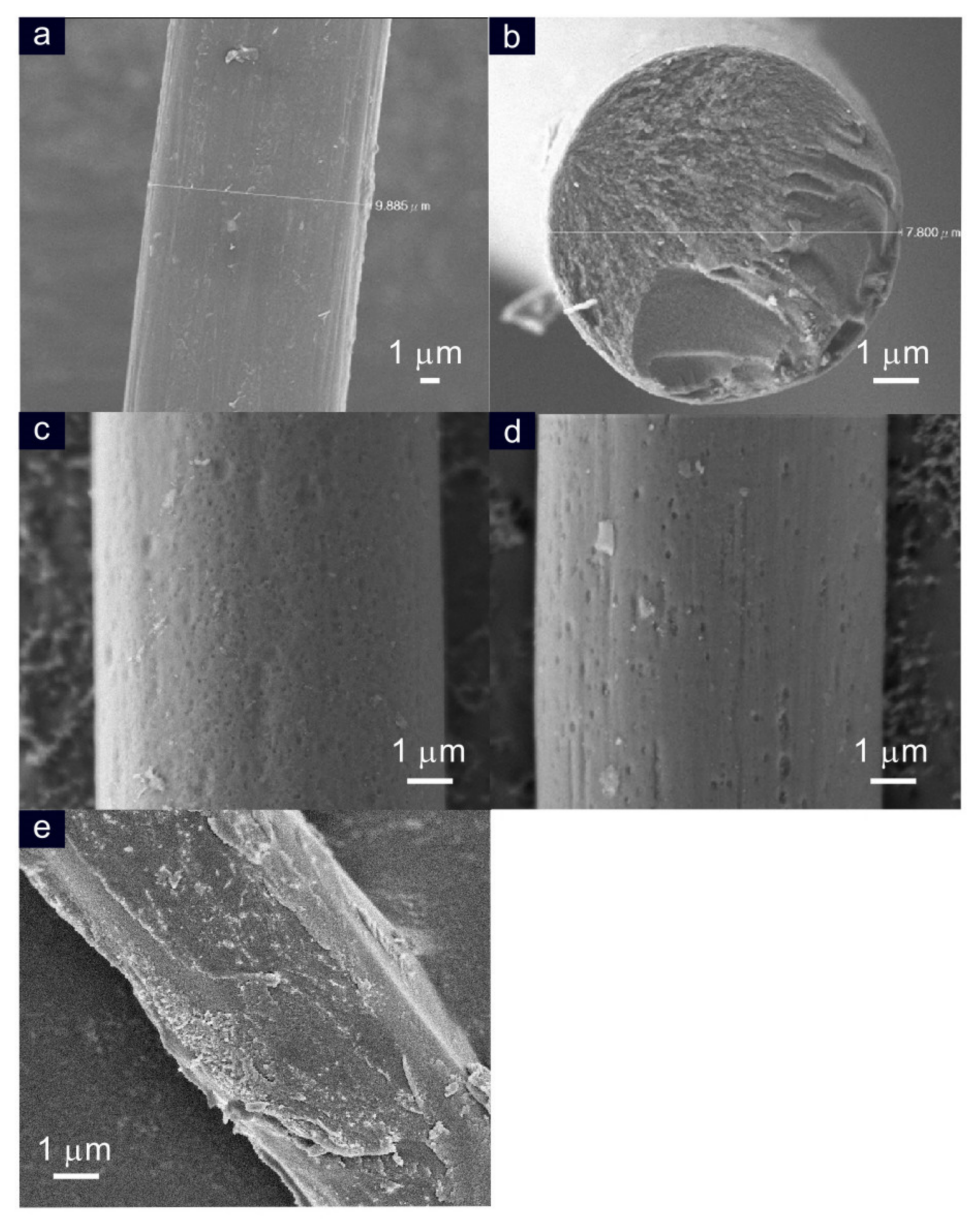
SEM morphology of carbon fiber before (**a**) and after the electro-exfoliated process performed in (**b**) 0.1 M of H_3_PO_4_, (**c**) 0.1 M PBS (pH = 7), (**d**) 0.01 M of NaOH, and (**e**) 0.01 M of NaOH + 0.1 M of PO_4_^3−^ solutions.

Figure 5a is an SEM image of carbon fiber before the electro-exfoliation experiment. A longitudinal fiber of diameter size 9.8 µm with a smooth morphology structure was noticed. Figure 5b–e are typical SEM images of the electrochemically exfoliated carbon fiber at various solution-phase conditions, such as 0.1 M of H_3_PO_4_ (b), 0.1 M pH 7 phosphate buffer solution (c), 0.01 M of NaOH (d), and 0.01 M of NaOH + 0.1 M of H_3_PO_4_ (e). A typical cross-sectional surface view of the carbon fiber conditioned at 0.1 M of H_3_PO_4_ is shown in Figure 5b. A porous interface morphology, which may be due to the exfoliation treatment, was noticed. Meanwhile, for the case of the 0.1 M PBS (pH = 7) conditioned system, a homogenous and minor porous surface structure like carbon fiber was observed (Figure 5c). This observation is true with the 0.01 M of NaOH system as well (Figure 5d). On the other hand, marked cracks with apparently deep grooves (leading to the rough surface) were found upon the electro-exfoliation of carbon fiber under the 0.01 M of NaOH + 0.1 M of PO_4_^3−^ solution (Figure 5e). Based on the previous literature relating to the solvent effect and external influencing conditions, it can be concluded that specific phosphate ion intercalation, temperature gradient, and electrochemical stress generated on the carbon fiber electrolyte interface are responsible factors for the facile electrochemical exfoliation of carbon nanoparticles as graphitic structures from the fiber [32,33] (Scheme 1A–C). Furthermore, it has been reported that hydroxyl and oxygen radicals that have been generated at a high oxidation potential (2 V vs. Ag/AgCl) (Scheme 1B) attack the graphite edge planes, facilitating the intercalation of PO_4_^3−^ anion with carbon nanofiber, and further contribute to the dissolution of hydroxylated carbon nanoparticles [11]. Since the 0.01 M of NaOH + 0.1 M of H_3_PO_4_ condition showed the best exfoliation process as compared to the other conditions, it was fixed as an optimal condition for further experiments.

**Figure 6:**
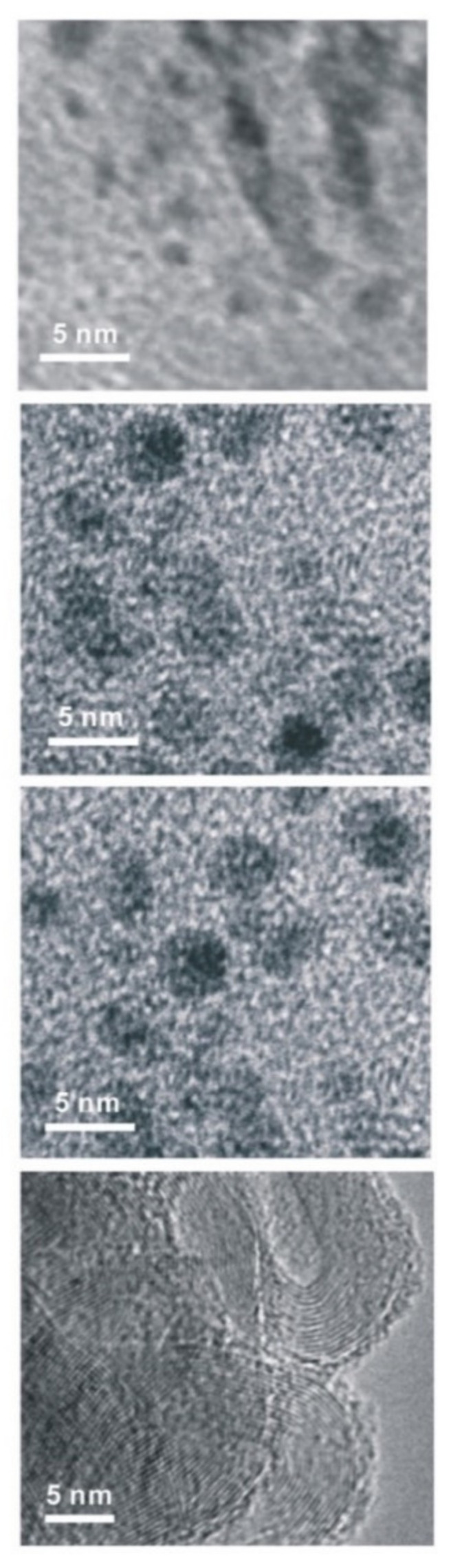
TEM images of carbon nanomaterials obtained by the electro-exfoliation reaction carried out at applied potential of 2.0 V versus Ag/AgCl in 0.01 M of NaOH + 0.1 M of PO_4_^3−^ solutions at various temperatures of 0 °C, 20 °C, 30 °C, and 40 °C (Top to bottom respectiverly).

To determine the information about the particle size, the as-prepared carbon nanoparticle samples were subjected to TEM and particle size analyses discreetly, as shown in Figure 6. Systematic growth of particle size from 1 nm to ~14 nm was noticed upon increasing the exfoliation temperature from 0 °C to 40 °C in 0.01 of NaOH + 0.1 M of PO_4_^3−^. Colloidal particles of uniform sphere-like morphology were observed when the exfoliation temperature was below 30 °C (Figure 6), whereas a clear, hollow spherical onion-like structure of carbon nanoparticles (Figure 6) was observed at 40 °C. Meanwhile, a decrement of approximately 2–3% in the diameter of the carbon fiber was noticed. It is likely that the slow generation of carbonaceous intermediate product formation [34] around the carbon fiber surface and the simultaneous carbonization reaction on the electrolyte interface is the reason for the onion-like structure appearance in this work. Based on the experimental results, the obtained sizes of the carbon particulate systems are 1.0 ± 0.1; 4.8 ± 1.5; 9.5 ± 2.8; and 13.3 ± 3.8 nm, respectively at exfoliation temperatures of 0; 20; 30; and 40 °C. An electrochemical exfoliation condition of electrolyte = 0.01 M of NaOH + 0.1 M of PO_4_^3^^−^ and temperature = 40 °C was identified as an optimal system for the synthesis of a clean and uniform-sized carbon nanoparticle. Note that when compared with other reports [9,35,36], a highly monodispersive morphology observed in this work is a major highlight.

**Figure 7:**
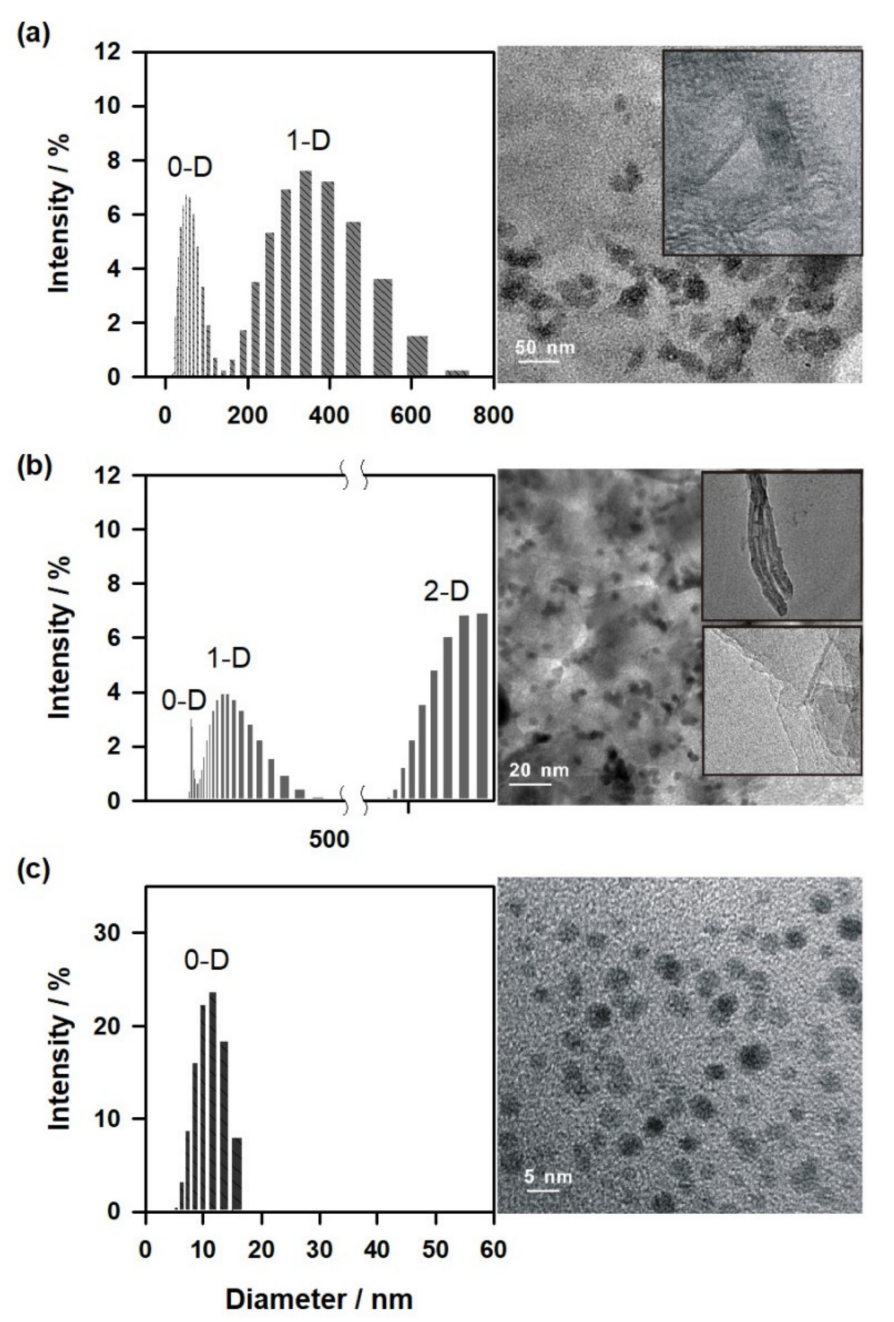
TEM images and size distribution of carbon nanomaterials obtained by the electrochemical exfoliation reaction carried out at an applied potential of 2.0 V versus Ag/AgCl in (**a**) 0.01 M of NaOH, (**b**) 0.1 M of PO_4_^3−^, and (**c**) 0.01 M of NaOH + 0.1 M of PO_4_^3−^ solutions at a fixed temperature of 40 °C.

To support the experimental observation, controlled electrochemical exfoliation experiments with individual electrolytes such as 0.01 M of NaOH, 0.1 M of PO_4_^3−^, and 0.01 M of NaOH + 0.1 M of PO_4_^3−^ were carried out at a fixed temperature of 40 °C, respectively. For the case of the 0.01 M of NaOH solution, two size distribution zones corresponding to sphere-like carbon nanoparticles and tube-like carbon nanoparticles were observed, respectively (Figure 7a). Similarly, for the case of the 0.1 M of PO_4_^3−^ condition, three size distribution zones were observed corresponding to sphere-like carbon nanoparticles, tube-like carbon nanoparticles (211.9 ± 37.7 nm), and thin graphites (i.e., multilayer graphene, 1136.2 ± 241.8 nm), respectively (Figure 7b). The experimental observation of mixed carbon nanoparticle formation is true for the majority of literature reports [37,38]. Plausibly, an uncontrolled method of electrochemical exfoliation reaction is the likely reason for the observation. Interestingly, sphere-like monodispersed carbon nanoparticles with a reduced particle size of 13.3 ± 3.8 nm were observed in the solution of 0.01 M of NaOH + 0.1 M of PO_4_^3−^ (Figure 7c). In connection with the observation, Toyoda’s group has reported the effect of PO_4_^3-^ in the chemical exfoliation of carbon fiber at 1000 °C [39]. It has been pointed out that the sp^2^-bonded carbon network, together with carbon–oxygen surface groups at the edges with the OH^−^ ions intercalated in the interlaminar space like carbon nanomaterials, was formed.

### 3.3. Surface Functional Group Analysis

**Figure 8:**
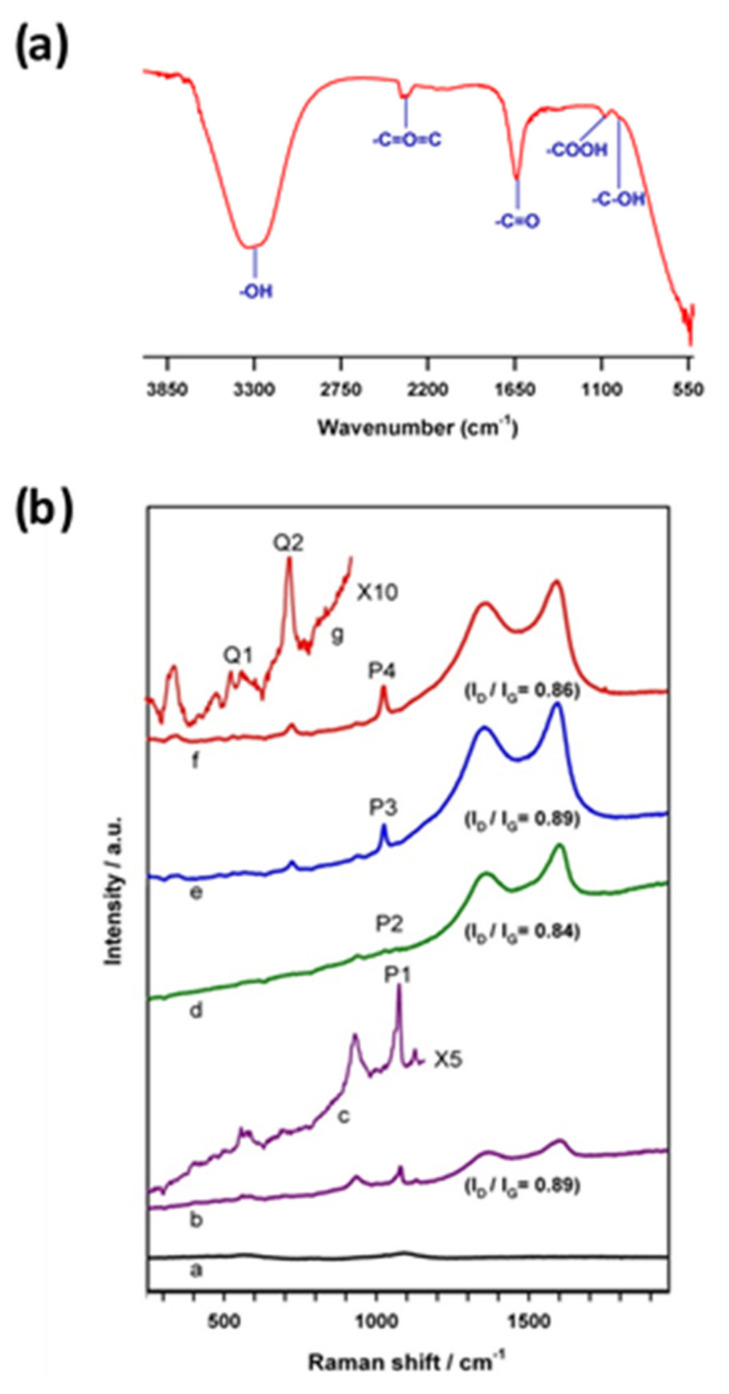
(**a**) FTIR of the electrochemically exfoliated carbon nanoparticles prepared at 0 °C. (**b**) Raman spectra of carbon nanoparticles coated on ITO substrate: a: bare-ITO, and the electrochemically exfoliated carbon nanoparticles prepared at various temperatures; b: 0 °C; c: 0 °C spectrum magnified by a factor of 5; d: 20 °C; e: 30 °C; and f: 40 °C; g: 40 °C spectrum magnified by a factor of 10 in 0.01 M NaOH + 0.1 M PO_4_^3−^ solutions. Applied potential, 2 V vs. Ag/AgCl.

Figure 8a is a representative FTIR response of CNQ prepared at 0 °C. Carbon–oxygen functional groups due to O–H stretching vibration (3200–3500 cm^−1^), -C=O group (1645 cm^−1^), -C–OH group (875 cm^−1^) and -COOH group (1051 cm^−1^) were observed. This observation denotes the existence of a rich surface oxygen functional group on the carbon nanomaterial.

Figure 8b is comparative Raman spectroscopic data of CNPs prepared at different temperatures. Qualitatively similar Raman patterns were noticed due to graphitic (D-band, sp^2^ carbon) and disordered graphitic (G-band, sp^3^ carbon) structures at frequencies of 1344 cm^−1^ and 1650 cm^−1^, respectively. The ratio of the intensities of D and G bands, I_D_/I_G_ is commonly used to estimate the degree of the perfection of the graphene structure [40]. It is expected that the electrochemical exfoliations led to a marked edge plane (a strong D peak) on the surface of carbon nanoparticles (as shown as I_D_/I_G_ ratios in Figure 8b(b–f)). Calculated I_D_/I_G_ values are 0.89, 0.84, 0.89, and 0.86, respectively. Nearly the same I_D_/I_G_ value noticed against the various preparation temperature may indicate a similar qualitative structure of the carbon nanoparticle prepared in this work. Indeed, the specific pre-peaks (T-peaks) noticed around 1026 cm^−1^ with high-temperature samples, which were due to the UV excitation peak, indicate intense graphitic structure formation with those samples [41,42].

**Figure 9:**
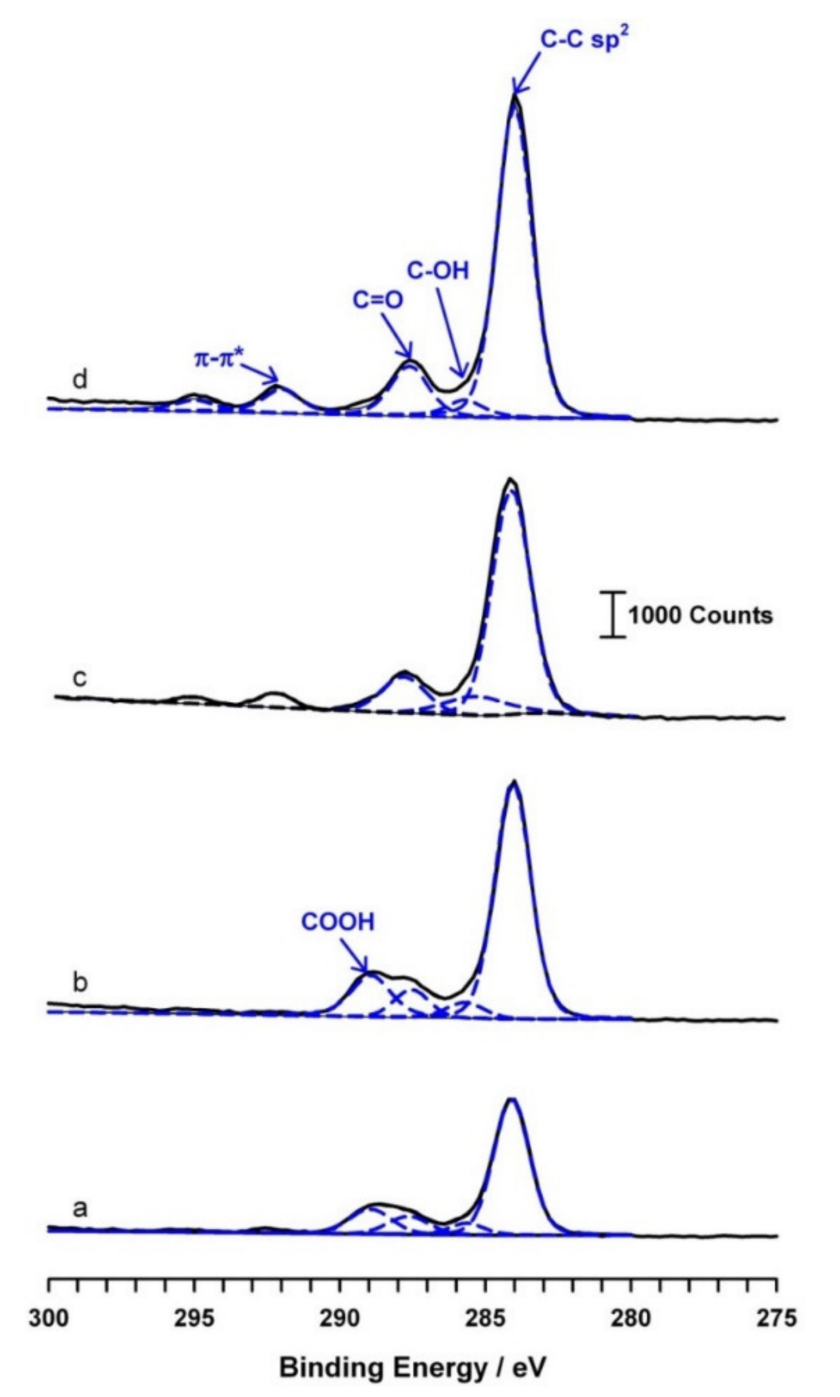
The XPS spectrum of the exfoliated carbon nanoparticles attained through electro-exfoliated at 2.0 V versus Ag/AgCl in 0.01 M NaOH + 0.1 M PO_4_^3−^ solutions at (**a**) 0 °C, (**b**) 20 °C, (**c**) 30 °C, and (**d**) 40 °C.

The XPS technique was adopted to precisely analyze the surface functional groups of the carbon nanoparticles that were prepared at various temperatures. Figure 9 shows the typical high-resolution C1-core level XPS of the carbon nanoparticles. By using a curve-fitting software program, individual carbon species were deconvoluted. A native carbon, >C< (284.4 eV) along with marked carbon–oxygen species like -C-OH (phenol/alcohol, 285.7 eV), -C=O (carbonyl, 288.0 eV), and -COOH (carboxylic, 289.4 eV) can clearly be seen with the 30 °C and 40 °C prepared samples. A similar kind of XPS spectra was observed in our previous study on the pre-anodized SPCE with an intense C1 (283.3 eV) response due to the polymer binder used, along with carbon–oxygen functional groups [22]. Indeed, a remarkable π–π* shake-up satellite peak at binding energy ~291.5 eV, a characteristic of aromatic or conjugated systems, was observed specifically with the samples prepared at 30 °C and 40 °C in this work (Figure 9c,d). However, with the sample prepared at 20 °C, there is no marked observation for the carboxylic group (-COOH) and the satellite peaks formation (Figure 9a,b). Temperatures >30 °C are likely a threshold condition for the electrochemical exfoliation reaction. Based on the physicochemical characterization results and size obtained in a window, 1–14 nm, it is revealed that the carbon nanoparticles formed in this work are graphene oxide quantum dots (GOQDs) structures. It is noteworthy that, unlike the literature [37,43], in this work, a simple and highly efficient electrochemical exfoliation technique has been introduced for the one-step synthesis of size-controlled GOQDs.

### 3.4. Electroanalytical Application

**Figure 10:**
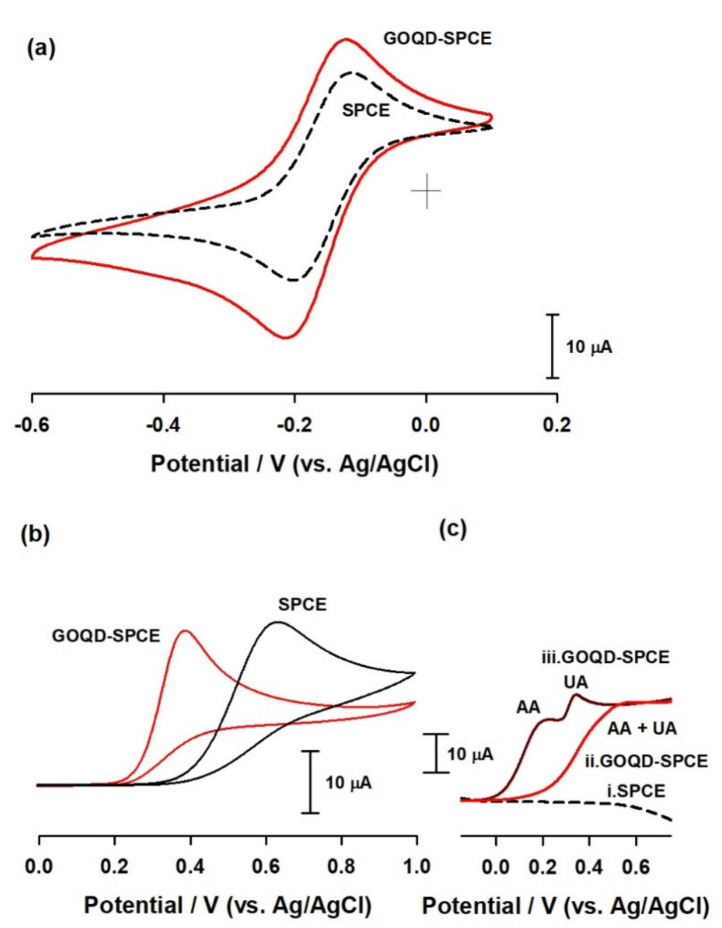
(**a**) Cyclic voltammograms of 1 mM [Ru(NH_3_)_6_]Cl_3_ at a bare SPCE and sphere-like GOQD-modified SPCE (GOQD-SPCE) in 0.1 M of NaH_2_PO_4_ solution at a scan rate of 50 mV/s. (**b**) Cyclic voltammograms of 1 mM of uric acid at a bare SPCE and GOQD-SPCE in 0.1 M, pH 8 PBS at a scan rate of 50 mV/s. (**c**) Linear scan voltammograms of SPCE (i) and GOQD-SPCE (ii) for simultaneous determination of ascorbic acid (AA, 1 mM) and uric acid (UA, 500 μM), and a control response of GOQD-SPCE without any analyte (iii).

To test the electroanalytical applicability, the GOQD sample was modified directly on a screen-printed carbon surface (SPCE) by drop-casting technique and extended to electrochemical studies. Initial CV experiments were carried out with a standard redox couple, Ru(NH_3_)_6_^3+^, which is usually used as an outer sphere probe, and as a functional group-insensitive compound for electrochemical systems [44]. As presented in Figure 10a, there was about a 20% enhanced peak current response on GOQD over respective control SPCE, which may be due to negative charge carried with GOQD and enhanced electronic conductivity effects. Next, simultaneous electrochemical determination of uric acid (UA) and ascorbic acid (AA) were chosen as a model compound due to their importance in a biological system [45,46]. In general, owing to their closer electrochemical oxidation potentials, an overlapped electrochemical oxidation signal was observed while analyzing these compounds. Figure 10c (top) is a typical CV response of simultaneous detection of AA and UA at an unmodified SPCE in pH 8 phosphate buffer solution showing a single peak-overlaid response. Thus, it is a challenging task to develop well-resolved electrochemical signals for the two biomolecules. Interestingly, when the same experiment was repeated with the GOQD-modified SPCE, GOQD-SPCE, well-defined and resolved peaks for the UA and AA were obtained (Figure 10b (bottom)). The negative shift of oxidation peak potential is the specific sign of the improved electrochemical performance of the modified electrode (Figure 10b). This observation is similar to the response noticed with a graphitic oxide-modified SPCE and better than that of the C_60_ fullerene-modified glassy carbon electrode performance [47,48]. It appears that the oxygen-containing functional groups can not only protect the carbon nanomaterials from aggregation, but also provide hydrogen bonding to enhance the electroanalytical activity (an inner-sphere electron-transfer mechanism).

### 3.5. Au-Nanoparticles Stabilized GOQDs

**Figure 11:**
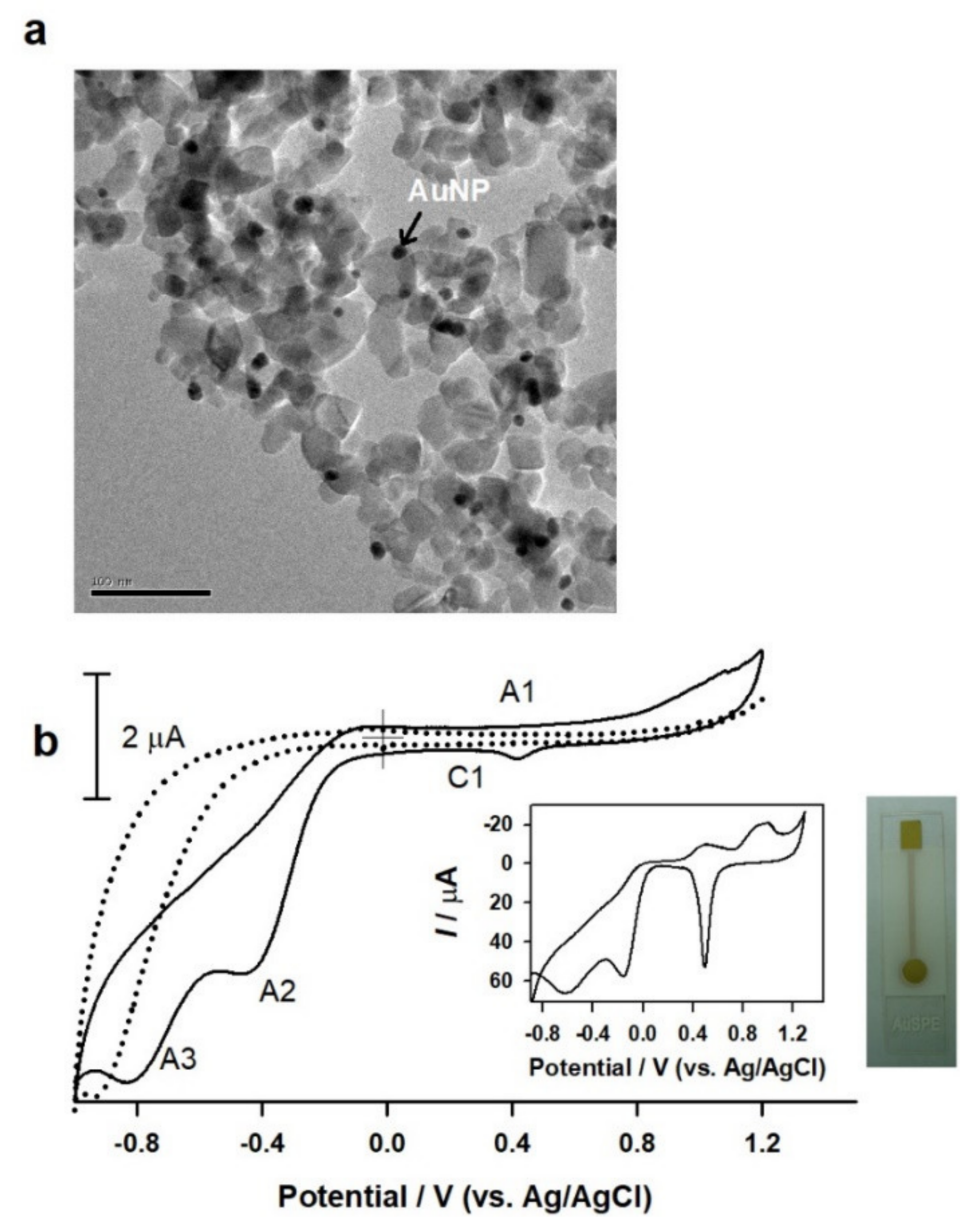
TEM image (**a**) and the observed cyclic voltammogram (**b**) of gold nanoparticles (AuNPs) stabilized CNP (GOQDs). Inset is a photograph of a commercial AuSPE that was used as a control in this work. Cyclic voltammograms of a bare SPCE (dotted-line) and vapor-deposition gold electrode (AuSPE, insert figure) in 0.1 M, pH 8 PBS at a scan rate of 50 mV/s are presented as controls.

Using GOQDs as a template, gold nanoparticles (AuNPs) with a narrow size were synthesized, as shown in Figure 11. In brief, a uniform-sized gold nanoparticle of GOQD (GOQDs@AuNPs) was prepared simply by mixing solutions of onion-sphere-like carbon nanoparticles and HAuCl_4_ (5:1, *v*/*v*) with methanol (as a reducing agent) at a temperature of 70 °C for 30 min under constant stirring. Figure 11a is a typical TEM image of the AuNPs@GOQDs showing black-colored dots of size ~2 nm embedded on the spherical carbon nanoparticle matrix. AuNP formation was confirmed by the EDX experiment (data not enclosed). The CV response of 9 µL AuNPs@GOQDs drop-coated on SPCE, SPCE/AuNPs@GOQDs in comparison with a control screen-printed gold electrode (AuSPE) are displayed in Figure 11b. A specific Au/Au_2_O_3_ (A1-peak) response at 0.4 V vs. Ag/AgCl and other shoulders like peaks at −0.4 (A2) and −0.65 V vs. Ag/AgCl (A3) due to successive 2e^−^ reduction of dissolved oxygen reduction responses mediated by the gold nanoparticles were observed. These observations confirm the formation of gold nanoparticles stabilized by GOQDs in this work. A similar kind of graphene oxide-Au-nanoparticle was prepared using a reduction of gold precursor, AuCl_4_^−^ by nitroarenes [49] and methanol (under UV-light irradiation) [50], and a deliberate mixture solution of Au-nanoparticle/Cetyltrimethylammonium bromide (CTAB) + GO [51]. In this work, the oxygen-rich functional groups like -COOH and -OH, might have been involved in the in situ reduction of the Au^3+^ ion to AuNPS formation. Thus, the “as-prepared gold nanoparticles” can be used as a platform for fast, sensitive, and selective detection of biomolecules. Research along this line is currently being carried out in our laboratory.

Overall, the present approach for the preparation of size-controlled graphene oxide and related carbon nanoparticles is simple, low-cost and does not involve any harsh or hazardous chemicals such as H_2_O_2_, concentric H_2_SO_4_, HNO_3_, or strong oxidants such as KMnO_4_. It is a safe and environmentally friendly approach for the future development of GO-based carbon nanoparticles for real-time applications [52].

## 4. Conclusions

Electrochemical exfoliation of PAN carbon fiber in alkaline condition at an applied voltage, 2V vs. Ag/AgCl with a set temperature of 40 °C has resulted in a facile formation of carbon nanoparticles. The effects of solution-phase and temperature conditions were systematically optimized. Adding phosphate ions in the alkaline state was found to enhance the appearance of uniform-sized carbon nanoparticles. Based on various physicochemical and spectroscopic characterization methods, the exfoliated carbon nanoparticles formed in this work have been identified as 14 ± 4 nm-sized carbon–oxygen-rich graphitic oxide-quantum dots (GOQD). Unlike the literature reports involving several off-line post-sample procedure treatments, a simple one-step and direct synthesis method has been reported for uniform-sized carbon nanoparticle preparation. When it is stored at room temperature, the GOQDs showed stability over a six-month period. As a utility to electroanalytical applications, simultaneous voltammetric sensing of uric acid and ascorbic acid with well-resolved peak potential separation has been demonstrated. Furthermore, utilizing the GOQDs as a template, a gold nanoparticle GOQDs composite system of average size ~2 nm has been synthesized and shown a stable electrochemical feature. Since the method of CNP preparation is direct and straightforward, several electroanalytical applications are ready to be imagined with this new system. Further application works are in progress.

## Abbreviations

Carbon nanoparticle-CNP, graphene-Gr, graphene oxide-GO, graphene quantum dots-GQD, graphene oxide quantum dots-GOQD, AuNPs-Gold nanoparticles.
